# Systemic neurophysiological entrainment to behaviorally relevant rhythmic stimuli

**DOI:** 10.14814/phy2.70079

**Published:** 2024-10-08

**Authors:** Manuel Muñoz‐Caracuel, Vanesa Muñoz, Francisco J. Ruiz‐Martínez, Antonio J. Vázquez Morejón, Carlos M. Gómez

**Affiliations:** ^1^ Department of Experimental Psychology University of Seville Sevilla Spain; ^2^ Mental Health Unit Hospital Universitario Virgen del Rocio Seville Spain; ^3^ Department of Personality, Evaluation and Psychological Treatments University of Seville Sevilla Spain

**Keywords:** embodiment, entrainment, oscillations, predictive processing, rhythm

## Abstract

Physiological oscillations, such as those involved in brain activity, heartbeat, and respiration, display inherent rhythmicity across various timescales. However, adaptive behavior arises from the interaction between these intrinsic rhythms and external environmental cues. In this study, we used multimodal neurophysiological recordings, simultaneously capturing signals from the central and autonomic nervous systems (CNS and ANS), to explore the dynamics of brain and body rhythms in response to rhythmic auditory stimulation across three conditions: baseline (no auditory stimulation), passive auditory processing, and active auditory processing (discrimination task). Our findings demonstrate that active engagement with auditory stimulation synchronizes both CNS and ANS rhythms with the external rhythm, unlike passive and baseline conditions, as evidenced by power spectral density (PSD) and coherence analyses. Importantly, phase angle analysis revealed a consistent alignment across participants between their physiological oscillatory phases at stimulus or response onsets. This alignment was associated with reaction times, suggesting that certain phases of physiological oscillations are spontaneously prioritized across individuals due to their adaptive role in sensorimotor behavior. These results highlight the intricate interplay between CNS and ANS rhythms in optimizing sensorimotor responses to environmental demands, suggesting a potential mechanism of embodied predictive processing.

## 1 INTRODUCTION

Oscillations are fundamental organizing phenomena in biological systems, providing functional advantages like predictive capabilities, energy‐efficiency, and enhanced communication through noise resilience and desensitization prevention (Glass, 2001; Rapp, 1987; Xiong & Garfinkel, 2023). In the human body, numerous physiological processes, including heartbeat, respiration, and neural activity, exhibit oscillatory behavior and often interact with external environmental rhythms, as seen in music, dance, language, sports, and other human activities that require adaptive responses to rhythmic patterns (Charalambous & Djebbara, 2023; Greenfield et al., 2021; Kotz et al., 2018).

Physiological oscillations vary across timescales, but adaptive behavioral control may rely on their temporal alignment with external inputs, facilitating efficient responses to environmental demands (Penzel et al., 2017; Pezzulo et al., 2015). In this context, when physiological oscillations synchronize in phase and/or frequency with an external oscillatory source, they are said to be entrained to that rhythm (Lakatos et al., 2019; Pikovsky et al., 2001). Entrainment has been proposed to underlie cognitive processes like selective attention by aligning neural phases of high excitability with periodic features of relevant external events, thereby enhancing neural gain for attended sensory inputs (Henry et al., 2014; Lakatos et al., 2008, 2013, 2019; Obleser & Kayser, 2019; Schroeder & Lakatos, 2009; Zion Golumbic et al., 2013). Neural entrainment has also been shown to improve behavioral performance, reducing reaction times, and increasing accuracy in auditory sensorimotor tasks (Nozaradan et al., 2016; Stefanics et al., 2010).

However, less in known about autonomic entrainment. While heart rate has shown inconsistent evidence of entrainment with rhythmic external stimuli (Mütze et al., 2020), respiration has been shown to entrain to auditory external stimuli that involve motor responses, though not during passive listening or with complex rhythms (Haas et al., 1986; Wilke et al., 1975). Respiration is recognized as an active sensory selection process modulating neural synchronization across widespread cortical regions to enhance information processing and cognitive performance (Brændholt et al., 2023; Herrero et al., 2018; Kluger et al., 2021; Maric et al., 2020; Perl et al., 2019; Zelano et al., 2016). Moreover, the breathing phase has been shown to systematically influence cognitive performance (Perl et al., 2019; Zelano et al., 2016), highlighting the bidirectional communication between body–brain signals aimed at adapting to task‐specific demands (Criscuolo et al., 2022).

Additionally, from the predictive processing perspective (Clark, 2013; Friston, 2010; Hohwy, 2013), the interplay between bodily and neural rhythms in response to external sensory inputs serves a predictive timing function. It aligns information sampling with bodily rhythms to minimize discrepancies between expected (preferred) and sensed body states through recurrent message passing among different hierarchical levels. This preserves organism integrity and supports adaptive goal‐directed behavior (Allen & Friston, 2018; Engel et al., 2001; Owens et al., 2018; Parr et al., 2022; Pezzulo et al., 2015; Seth & Friston, 2016).

Despite evidence of neural entrainment with periodic external events and the role of respiration in brain function, the intricate interplay between brain–body rhythms and external inputs remains poorly understood. Active engagement with sensory stimuli is believed to optimize bodily states for higher‐level information processing (Criscuolo et al., 2022; Parviainen et al., 2022), but integrated experimental findings using multimodal neurophysiological measures are scarce. Moreover, most studies exploring bodily entrainment to external stimuli have predominantly utilized simple repetitive stimuli to assess temporal predictive abilities. Incorporating experimental designs that account for the inherent complexities of discriminatory sensorimotor tasks could significantly contribute to understanding the multifaceted dynamics governing brain–body interactions with rhythmic cues.

This study aims to shed new light on the complex physiological dynamics of brain and body rhythms in response to rhythmic auditory stimulation, and to elucidate their behavioral implications by differentiating between passive and active sensory encoding processes. Simultaneous recordings of the central and autonomic nervous systems (CNS and ANS) were used during an experimental paradigm featuring temporally invariant rhythmic stimulation. Distinct auditory sequences were presented to dynamically modulate attentional demands on a trial‐by‐trial basis during a cueing task, fostering adaptive speed‐accuracy responses that better reflect dynamical sensorimotor contexts. We hypothesize that active engagement with rhythmic auditory stimuli will lead to greater entrainment of CNS and ANS physiological rhythms to the external rhythm, compared to passive listening and baseline conditions. Furthermore, during the active condition, we expect higher accuracy and reduced reaction times for the more prevalent phases of bodily oscillations at the target onset.

## 2 MATERIALS AND METHODS

We employed a systemic approach to examine various brain and body neurophysiological measures in response to rhythmic auditory stimulation under different attentional conditions. Our analysis included power spectral analysis and phase coherence to compare the alignment of physiological oscillations with the external rhythm across conditions. In addition, we used circular statistics and linear mixed models to explore detailed patterns and their behavioral implications.

### 2.1 Participants

A sample of 32 healthy participants (12 males and 20 females, 29 right‐handed and 3 left‐handed) aged between 19 and 36 years old (mean = 28.31 ± 3.87 SD) were recorded. Two participants were excluded from the analysis due to technical issues during the EEG recording. The study was approved by the Bioethical Committee of the Junta de Andalucía. Participants did not report any neurological disease or auditory impairment. The experiments were conducted with the informed and written consent of each participant, following the Helsinki Protocol.

### 2.2 Signal acquisition

**FIGURE 1:**
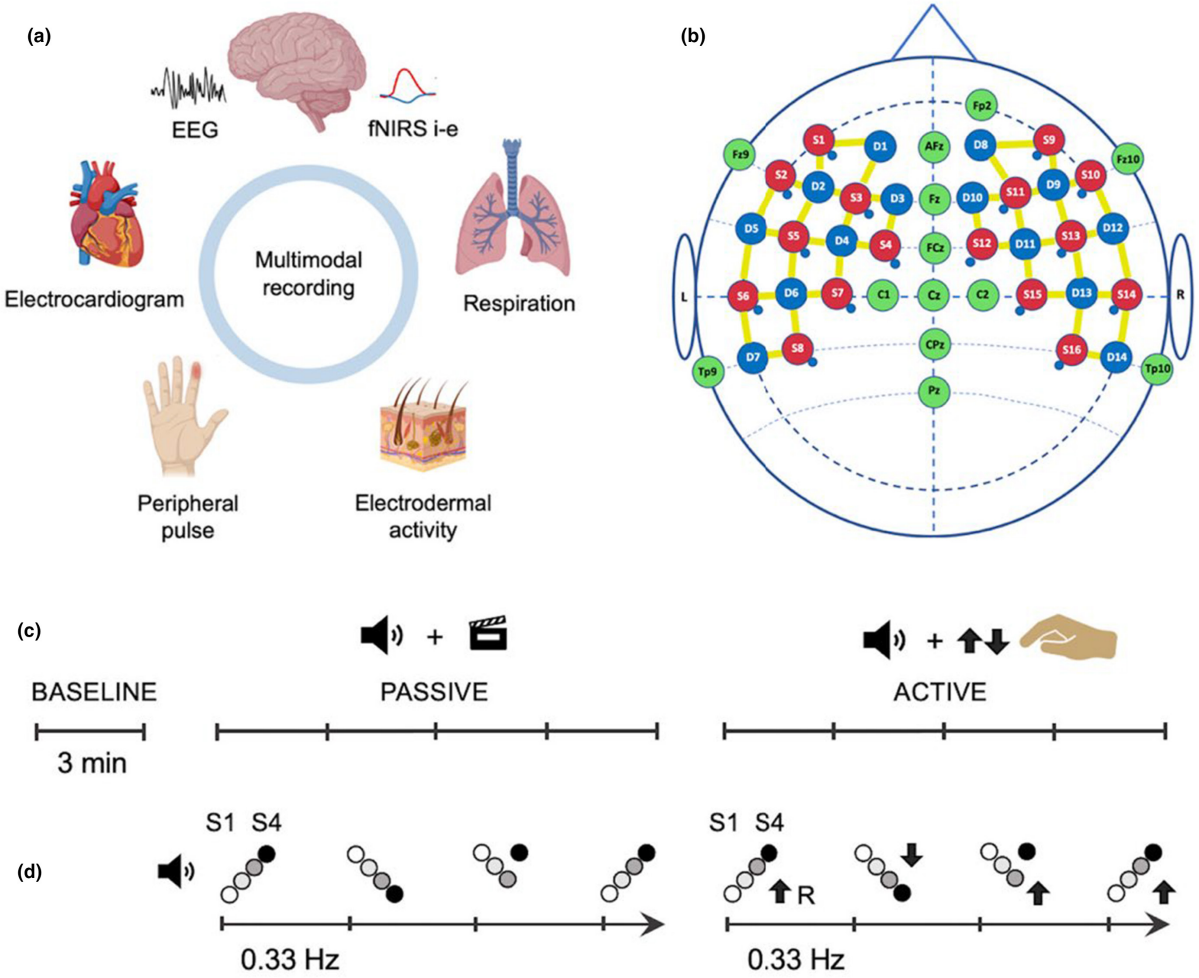
(a) Multimodal signal acquisition representation (created in BioRender.com). (b) Probe layout of fNIRS optodes and EEG electrodes. Red circles indicate fNIRS sources, blue circles fNIRS detectors, and green circles EEG channels. Yellow linear segments between sources and detectors indicate fNIRS standard separation channels. Overlapping small blue circles indicate the location of fNIRS short separation channels. (c) Graphical representation of the experimental design. Three conditions: Baseline, Passive (auditory stimuli + muted film) and Active (auditory stimuli + pressing either the up or down arrow of the keyboard). (d) Auditory stimulation exemplification for the passive and active condition, respectively. S1 and S4 indicate the first (cue) and fourth stimulus (target), respectively, of each auditory sequence. Linear auditory sequences were occasionally broken (20% incongruent trials) in the last stimulus (S4). In the active condition, the expected responses after the arrival of S4 are indicated by black arrows, resulting from the comparison of the auditory frequency of S4 with that of the preceding stimulus (S3) within each trial.

A multimodal signal acquisition was performed, covering different measures of the central and peripheral nervous system at the same time (Figure 1a). Electroencephalography (EEG) and intracerebral Functional Near Infrared Spectroscopy (fNIRS‐i) recorded the brain electrical activity and oxygenation changes, respectively. Electrodermal activity (EDA), Electrocardiogram (ECG), Plethysmography of the Peripheral Pulse (PPG), Respiratory effort (RSP), and extracerebral Functional Near Infrared Spectroscopy (fNIRS‐e) were recorded to assess physiological peripheral signals controlled by Autonomic Nervous System (ANS) activity.

#### 2.2.1 EEG

The EEG was recorded using active electrodes (ActiCAP) from 13 scalp sites mainly distributed along the midline (Figure 1b) with a Brain Vision V‐Amp DC amplifier (Brain Products, Munich, Germany). The electrode impedance was kept below 20 kΩ. The left mastoid was used as the reference. DC amplification gain was 20,000, and the sampling rate was 1000 Hz. Data acquisition was done using BrainVision Recorder 1.20 (Brain Products).

#### 2.2.2 fNIRS

The fNIRS signal was recorded using a NIRScoutXP device (NIRx Medical Technologies, Glen Head, NY, USA) with 16 LED sources and 30 detectors (14 standard separation detectors +16 short separation detectors) placed along the fronto‐temporal areas of both sides of the scalp (Figure 1b), obtaining a total of 58 channels (42 standard separation channels, 16 short separation channels). Intracerebral (cortical) oxygenation changes (fNIRS‐i) were captured by the standard separation channels, whereas extracerebral oxygenation changes (fNIRS‐e) on the scalp surface were captured by the short separation channels. The source‐detector distance was 3 and 0.8 cm for the standard and the short separation channels, respectively. The sampling rate was 3.91 Hz. Data acquisition was accomplished with NIRStar v.14.2 software.

#### 2.2.3 Peripheral signals

The autonomic peripheral signals were recorded using an MP160 (BIOPAC Systems, Goleta, CA, USA) with four amplifier modules (PPG‐100C, EDA‐100C, ECG‐100C, and RSP‐100C), to obtain the PPG, EDA, ECG, and RSP signals, respectively.

The peripheral pulse was recorded through a plethysmograph (TSD200 transducer with a wavelength of 860 nm ± 60 nm‐ infrared light) placed on the index finger of the left hand. EDA was obtained using bipolar Ag‐AgCl finger electrodes placed on the ring and middle fingers of the left hand. ECG was acquired using three Ag‐AgCl lead electrodes (positive, negative, and ground, TSD203 transducer) placed on the left wrist, right wrist, and right ankle, respectively. RSP was recorded through a transducer band placed on the chest (TSD201 transducer), which allows measurement of changes in thoracic circumference that occur with breaths. The amplification gain was set as 100 in PPG, 5 in EDA, 1000 in ECG, and 10 in the RSP amplifiers. The sampling frequency was 1000 Hz. Data acquisition was performed using AcqKnowledge v.5.0.1 software (BIOPAC Systems).

### 2.3 Experimental paradigm

Participants were comfortably seated in a quiet room in front of a table equipped with a screen and a computer keyboard. The experiment was divided in three consecutive stages or conditions: baseline, passive and active condition (Figure 1c). During the baseline condition, participants were asked to stay as quiet as possible for 3 min, looking at a fixed cross in the middle of the screen without any other requirement. In the passive and the active conditions, the same auditory stimulation was used (Figure 1d). The stimuli consisted of trials of four consecutive pure auditory tones, presented in an ascending or descending frequency pattern (50%–50%, randomly counterbalanced within participants) that was occasionally broken (incongruent trial) in the last tone of the trial (S4). The auditory frequencies used for the tones were 600, 900, 1200, and 1500 Hz. Each tone lasted 200 ms (with a 20 ms rise‐fall time) and the inter‐stimulus interval (ISI) was 100 ms, resulting in 1100 ms of auditory stimulation, which was followed by 1900 ms of silence, comprising a total of 3000 ms (0.33 Hz) within each trial. Each condition consisted of 240 trials, with an 80%–20% ratio between congruent and incongruent trials, respectively. The experiment was programmed using PsychoPy v.2021.1.2 free software package (Peirce, 2009). The auditory stimuli were edited using Audacity free software package, v.3.0.0.

Both the passive and active conditions lasted 12 min and shared the same auditory paradigm, but the task instructions in each condition were different. In the passive condition, the participants were asked to watch a muted film and ignore the auditory tones, whereas in the active condition the participants were asked to pay attention to the tones and to respond in each of the trials by pressing the up or down arrow button on the keyboard, depending on whether the fourth auditory tone of the sequence (S4, target stimulus) had a lower or higher auditory frequency than the previous one (S3), trying to combine accuracy and speed in their responses (within a 1900 ms time window before the next trial).

The active task was divided into four blocks of 3 min (60 trials), separated by a short break, to prevent the effects of fatigue. There was a training period with an explanatory video, followed by a test trial with visual feedback. The order of conditions, first passive and then active condition, was intentionally kept constant across participants to avoid possible interference or conditioning factors from the active to the passive condition in the auditory processing system. Auditory stimuli were presented through two speakers (Dell, model A215), positioned on either side of the participant's head, at a comfortable volume of 60 dB (measured with a Velleman‐DVM1326 level sound‐meter).

### 2.4 Signal preparation and preprocessing

#### 2.4.1 EEG

EEG recordings were imported into EEGLAB v.14.1.1 and MATLAB 2022a software. To correct the EEG for eye blinking, ocular movements and muscle artifacts, an Independent Component Analysis (ICA) was computed. These components were removed, and the EEG signal was posteriorly reconstructed (mean = 3.5 ± 0.93 SD, range 2–7). The signal amplitudes in the electrodes Fp2, Fz9, and Fz10 were used to detect eye blink artifacts and ocular movements, while the 6 channels in the midline (AFz, Fz, FCz, Cz, CPz, and Pz) were the ones accounted for subsequent analysis.

#### 2.4.2 fNIRS

fNIRS raw data were imported into HOMER2 v.2.8 toolbox (Huppert et al., 2009) and MATLAB 2022a software. For the reduction of the signal motion artifacts, the function *hmrMotionCorrectionWavelet* was applied, with an interquartile range of 1.5. This function has proven to be quite robust in decreasing motion artifacts through wavelet decomposition (Brigadoi et al., 2014; Cooper et al., 2012; Molavi & Dumont, 2012). Additionally, the *enPCAFilter* function was applied, with one principal component being removed from the data (mean explained variance of the removed first component: 37% ±14%). The function *hmrSSR* was also applied, which regresses the activity in the short separation channels from the activity of the standard channels to eliminate the contribution of the extracerebral signal from the intracerebral (cortical) signal. The oxyhemoglobin (HbO) concentration changes were obtained employing the modified Beer–Lambert law with a differential partial pathlength factor of 6.0 for 760 nm and 5.0 for the 850 nm wavelength (F. Scholkmann et al., 2010). 3D brain images of the fNIRS channels layout were extracted with the AtlasViewer toolbox (Huppert et al., 2009).

#### 2.4.3 Peripheral signals

Peripheral signals were imported into AcqKnowledge v.5.0.1 (BIOPAC Systems), Ledalab (Benedek & Kaernbach, 2010) and MATLAB 2022a software. Signals were visually inspected to check that they were mostly free of movement artifacts. The skin conductance response (SCR) from the EDA signal was extracted with the Ledalab software (Benedek & Kaernbach, 2010) using the Continuous Decomposition Analysis (CDA) function. HR was extracted with the AcqKnowledge software from the R peaks detection of the ECG signal. The ECG and PPG signals were used to calculate the Pulse Transit Time (PTT), a proxy measure of the arterial pressure that can be calculated from the time interval between the R‐wave and the peak of the pulse wave at the finger. RSP and PPG raw signals were directly extracted from AcqKnowledge. Respiratory rate (RSP rate) was extracted from the RSP peak detection in AcqKnowledge.

All data, except for fNIRS, were downsampled to 100 Hz and detrended by subtracting a best‐fit third order polynomial. The HR and PTT tachograms were additionally smoothed using the *smooth*. *m* function of MATLAB with the ‘loess’ method, applying a smoothing parameter of 0.01. Data from the passive condition were artificially segmented into four blocks, aligning them with the structure of the active condition for comparative analysis. Consequently, a total of nine blocks (comprising one baseline, four passive and four active conditions), each consisting of 3 min of continuous data, were extracted for each signal and participant for subsequent data analysis.

### 2.5 Power spectral density

Power spectral analysis for all different signals, participants, blocks and channels (in the case of EEG and fNIRS) was performed using a custom MATLAB script. First, the data matrices were zero‐padded by a factor of 10 to increase the frequency resolution of the spectral analysis. Following zero‐padding, the FFT was applied to the data. From the FFT result, the one‐sided absolute values were obtained, since the FFT yields a complex result with both positive and negative frequencies. These absolute values were then squared and normalized by the sampling frequency and number of data points to obtain the power spectral density (PSD). Finally, the resulting power was doubled, except for the DC and Nyquist frequencies.

To compare conditions with absolute power differences and focus on relative power changes, the PSD results were normalized dividing the value at each frequency point by the total power. The PSD results of the four data blocks in both passive and active conditions were averaged, resulting in a single PSD signal per condition and participant, covering frequencies from 0 to the Nyquist frequency (50 Hz). Finally, to capture the spectral power at the frequency of interest (auditory stimulation at 0.33 Hz) and compare it between conditions, the integral of the PSD closely around this frequency (0.33 ± 0.005 Hz) was calculated for each condition and participant and exported for statistical analysis.

### 2.6 Phase coherence

Phase Coherence analysis between all recorded signals and the auditory stimulation for each participant, block, and channel (in the case of EEG and fNIRS) was performed using a custom MATLAB script, enabling the measurement of the consistency of phase differences over time. First, all data matrices were resampled to a common frequency rate of 20 Hz. Then, a zero‐phase 8th order bandpass IIR filter between 0.2–0.5 Hz was used. The reason for using this frequency range was to capture the frequency of interest (auditory stimulation at 0.33 Hz) at the same time that a potential confounder such as the respiratory frequency band (commonly reported around 0.15–0.5 Hz) containing it. The commonly used lower limit of 0.15 Hz for the respiratory frequency band was not used because it exceeded half the frequency of interest (0.33/2 = 0.165 Hz) and could introduce some harmonics that could alter the phase analysis. To enable the computation of phase coherence between physiological signals and stimuli, a cosine wave at 0.33 Hz was generated from the onset of auditory stimulation in each block. This cosine wave was then included as an additional signal for coherence analysis, representing the auditory stimulation. The continuous analytic signal of all data signals was obtained through the Hilbert transformation, and the phase angle was computed from these signals. Then, the phase difference between each pair of data signals were obtained, converted into complex space through the Euler's formula (eⁱ^x^ = cos x + i sin x), averaged, and transformed to absolute values to finally obtain the phase coherence between all pair of signals, ranging its value from 0 (no coherence) to 1 (maximum coherence). Finally, the coherence results from the four data blocks in both passive and active conditions were independently averaged to obtain a single value for each condition, facilitating a direct comparison among the baseline, passive, and active conditions. Likewise, coherence results from all EEG, fNIRS‐i and fNIRS‐e channels were also averaged for each data signal separately.

### 2.7 Phase angle

As an additional analysis to explore the specific contribution of distinct phases of the recorded signals to the phase coherence results in the active condition, the phase angles at the arrival of every first stimulus (S1, cue stimulus), fourth stimulus (S4, target stimulus), and response (R), as the drivers of interest, were extracted from the Hilbert transform of the preprocessed signals for each participant, converted to complex space through the Euler's formula and averaged across trials. The resultant mean value of the phase angles for each signal, participant and type of driver (S1, S4, and R) was then exported for subsequent statistical analysis. In the case of EEG, the FCz electrode was the one accounted for phase angle analysis as a central, equidistant, and relatively artifact‐free reference point. In the case of fNIRS‐i, the channel between source number 14 and detector number 13 (Figure 1b), on the right superior temporal gyrus, was the one considered due to the auditory nature of the experiment, whereas in the case of fNIRS‐e, the short separation channel adjacent to source number 14 (Figure 1b), on the right temporal region, was the one exported for phase angle statistical analysis.

Complementarily, to analyze potential linear‐circular relationships between reaction times and phase angles, these measures were extracted for each trial, type of trial (congruent or incongruent S4), participant and recorded signal. Subsequently, sine and cosine transformations were applied to the circular variable (phase angles), which allows to capture the periodic nature of the circular variable for statistical analysis. The relationship of hits and errors with phase angles was not finally analyzed due to the marginal existence of errors in the sample, as described in the results section.

### 2.8 Statistical analysis

Statistical analyses were carried out using SPSS 29.0.1 and R 4.3.2 (R Core Team, 2023) software packages. We first tested the normality of data distribution using a Shapiro–Wilk test and, when the normality assumption was violated, we applied appropriate nonparametric tests. To assess statistical differences between the processed signals, repeated‐measures analyses of variance (ANOVA) were performed with *condition* as a factor with three levels: baseline, passive and active. Greenhouse–Geisser correction was used when sphericity was violated. For EEG, fNIRS‐i and fNIRS‐e, the channels were included as a factor, in addition to condition. Post‐hoc contrasts (two‐tailed paired t‐test) were carried out when ANOVA differences reached significant results (*p* < 0.05). To assess the circular uniformity between phase angles of participants for each type of driver (S1, S4, and R) and processed signal, the Rayleigh's test (Jammalamadaka & SenGupta, 2001) was performed using the *circular* package implemented in R. A linear mixed‐effects (LME) model was utilized, using the *lmer* function in the *lmerTest* and *lmer4* packages implemented in R, to examine the association between reaction times and sine/cosine‐transformed phase angles, accounting for potential participant‐specific variability. The model included reaction time as the dependent variable, with sine and cosine‐transformed phase angles and type of trial (congruent/incongruent) as fixed effects. Additionally, a random intercept was incorporated for each participant to account for individual differences in reaction time. In all cases, the *p* values were adjusted for multiple comparisons when necessary by applying the False Discovery Rate (FDR) correction using the Benjamini‐Hochberg procedure.

## 3 RESULTS

### 3.1 Behavioral task

In the active condition task, the average hit rate was 236 out of 240 (98%) ± 5.54 SD, with half of the sample committing either just one error or none. Therefore, further analysis regarding accuracy performance relationship with phase angle could not be conducted. The mean reaction time was 692 ms (667 ms for congruent trials; 764 ms for incongruent trials) ± 202 ms SD. Differences in reaction time between type of trials reached significant results [t (1, 31) = 7.27; *p* < 0.001].

### 3.2 Power spectral density

**FIGURE 2:**
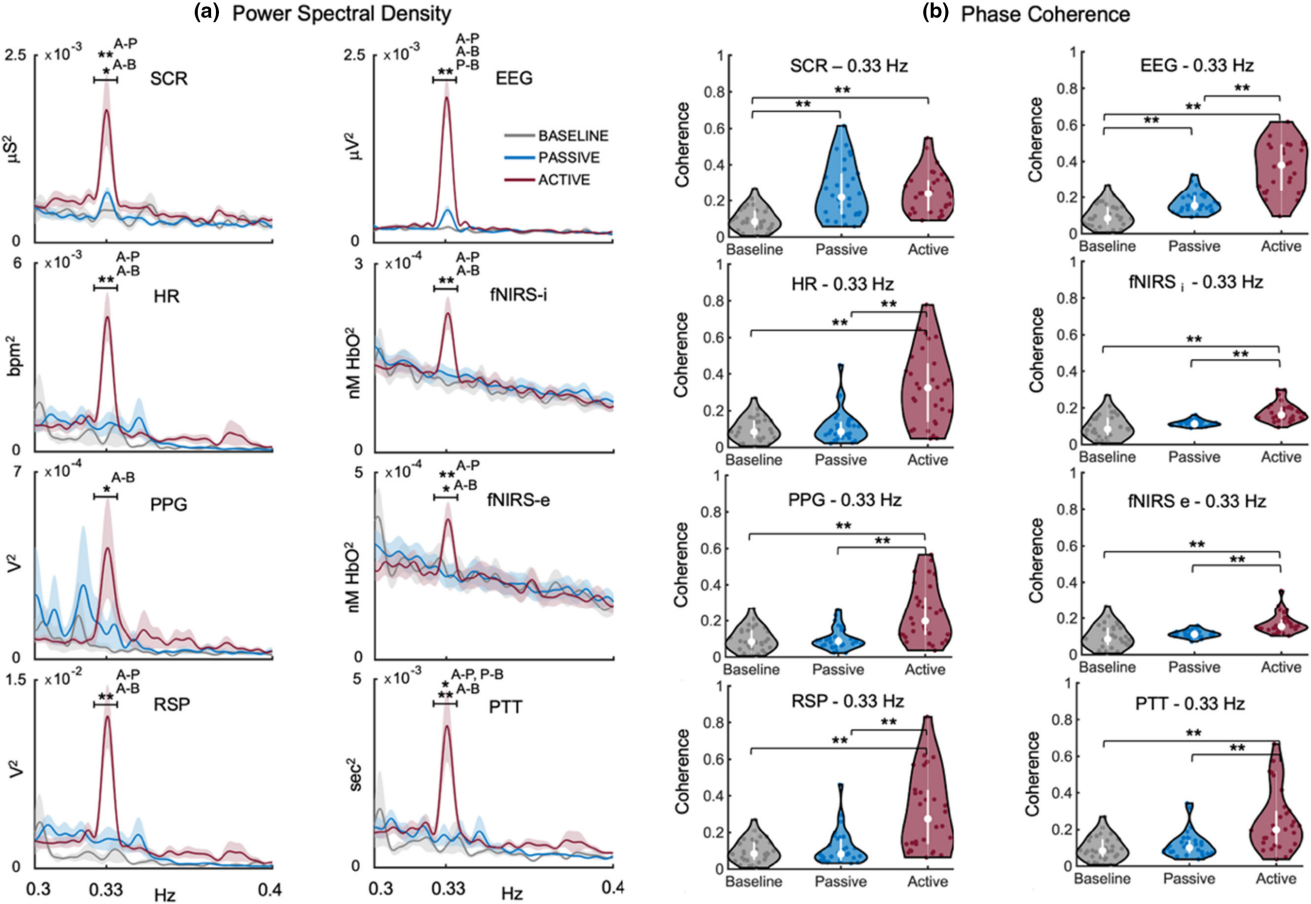
(a) PSD for the three experimental conditions (baseline, passive, and active) and the different data signals. The upper bounded line represents the accounted frequency window for statistical analysis. (b) Violin plots of the phase coherence between the different data signals and the auditory stimulation at 0.33 Hz for the three experimental conditions. For EEG, fNIRS‐i, and fNIRS‐e, the plotted PSD and phase coherence was the average of all channels. Vertical white lines inside the violins indicate the interquartile range, whereas the white point indicates the median value. Each colored point inside the violins represents a participant (*n* = 30). Significant differences between conditions are specified with an asterisk for *p*_(FDR)_ < 0.05 and two asterisks for *p*_(FDR)_ < 0.01. A, active condition; B, baseline condition; EEG, electroencephalographic activity; fNIRS‐e, extracerebral HbO; fNIRS‐i, intracerebral/cortical HbO; HR, heart rate; P, passive condition; PPG, peripheral pulse; PTT, pulse transit time; RSP, respiratory effort; SCL, skin conductane level.

The PSD for the different data signals around the frequency of interest (0.33 Hz) is shown in Figure 2a. The repeated‐measures ANOVA found significant statistical differences between conditions for all the recorded signals (Table 1).

**TABLE 1:** Statistical results from the PSD analysis around the frequency of interest (0.33 Hz).

Signal	*df*	*F*	*p*	*η* ^2^
SCR	1.236, 38.309	7.418	0.006	0.193
HR	1.400, 43.403	10.068	0.001	0.245
PPG	1.403, 43.487	3.586	0.051	0.104
RSP	1.279, 39.639	11.636	< 0.001	0.273
PTT	1.023, 31.710	7.659	0.009	0.198
fNIRS‐e	1.426, 44.195	7.601	0.004	0.197
fNIRS‐i	1.362, 42.234	14.172	< 0.001	0.314
EEG	1.088, 33.741	39.592	< 0.001	0.561

*Note*: Post hoc comparison analysis (FDR corrected for 24 comparisons: 3 conditions × 8 signals) revealed that the PSD of SCR, HR, RSP, EEG, fNIRS‐i, fNIRS‐e, and PTT around 0.33 Hz were higher for the active as compared with the passive condition [t (1, 29) = 2.546, 3.289, 3.149, 6.544, 3.509, 3.469, and 2.645, respectively; *p*_(FDR)_ = 0.016, 0.003, 0.004, <0.001, 0.001, 0.002, and 0.013, respectively], as well as for the active as compared with the baseline condition [t (1, 29) = 3.093, 3.430, 3.872, 6.759, 4.308, 2.422, and 2.890, respectively; *p*_(FDR)_ = 0.004, 0.002, <0.001, <0.001, <0.001, 0.021, and 0.007, respectively]. The PSD of PPG was greater for the active as compared with the baseline condition [t (1, 29) = 2.056; *p*_(FDR)_ = 0.048]. The PSD of EEG and PTT was also higher for the passive as compared with the baseline condition [t (1, 29) = 3.333, 2.218, respectively; *p*_(FDR)_ = 0.002, 0.034, respectively]. Altogether, these results indicate a significant amplitude peak at 0.33 Hz across all recorded signals during the active condition.

Abbreviations: *df*, degrees of freedom; EEG, electroencephalographic activity; *F*, F‐statistic; fNIRS‐e, Extracerebral HbO; fNIRS‐i, Intracerebral/cortical HbO; HR, heart rate; *p*, *p*‐value; PPG, peripheral pulse; PTT, pulse transit time; RSP, respiratory effort; SCR, skin conductance response; *η* ^2^, eta squared (effect size).

**FIGURE 3:**
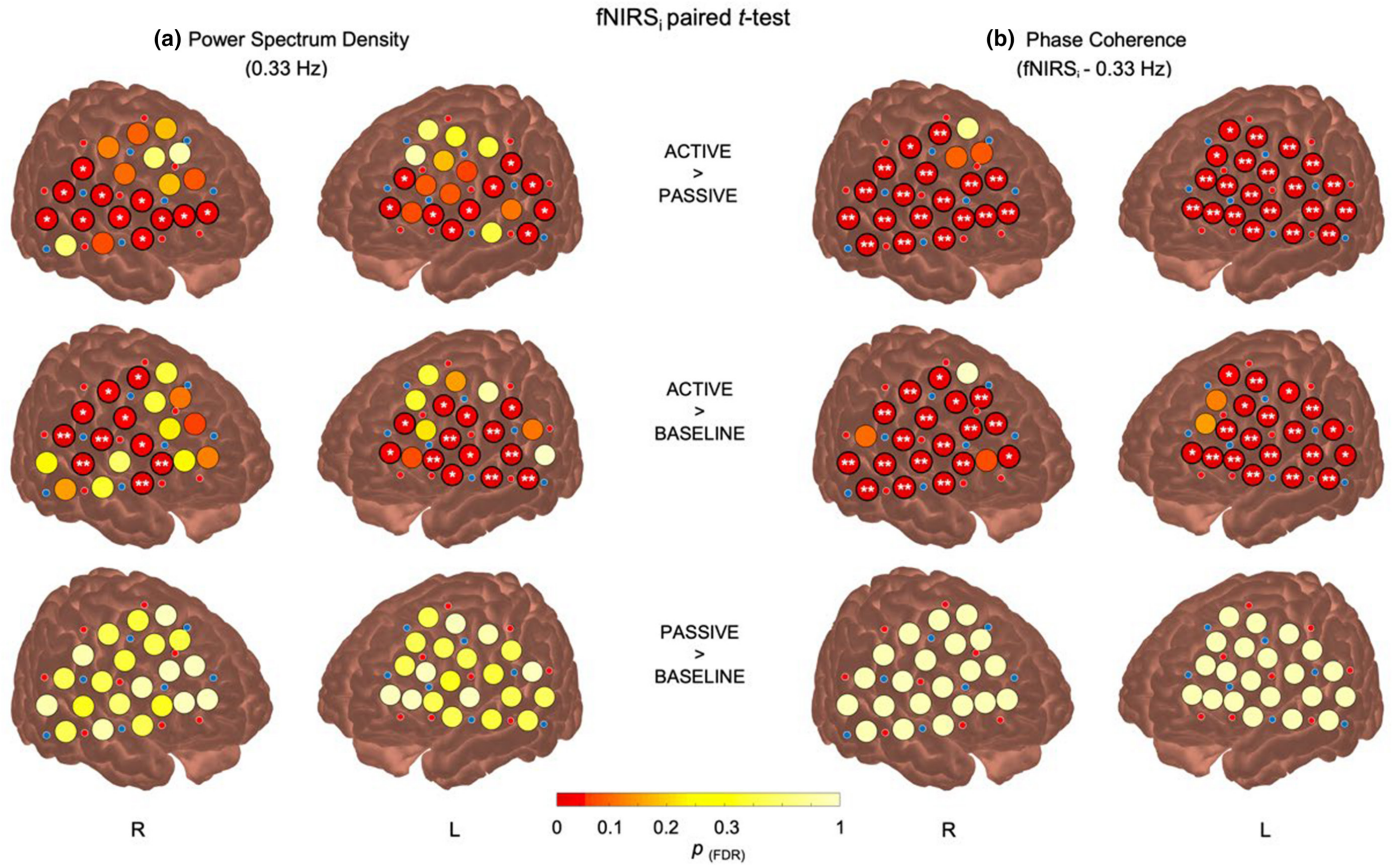
*p*‐values from *t*‐tests comparing pairs of conditions (top: Active > passive; middle: Active > baseline; bottom: Passive > baseline) for: (a) PSD of HbO levels for all fNIRS‐i channels and participants at 0.33 Hz. (b) PC between the HbO signal of all fNIRS‐i channels and the cosine signal at 0.33 Hz (representing the auditory stimulation) for all participants. The color gradient of the circles indicates the *p*_(FDR)_ values from the paired *t*‐test for each channel, according to the bottom color bar. Significant differences between conditions are specified within channels with an asterisk for *p*_(FDR)_ < 0.05 and two asterisks for *p*_(FDR)_ < 0.01. Red and blue circles on the brain surface represent the layout of fNIRS‐i sources and detector optodes, respectively.

The additional repeated‐measures ANOVA, incorporating channels as a factor, revealed a significant main effect of channels on the PSD around 0.33 Hz for fNIRS‐e [F (6.029, 186.902) = 5.813; *p* < 0.001; *η* ^2^ = 0.158], fNIRS‐i [F (6.644, 205.964) = 7.462; *p* < 0.001; *η* ^2^ = 0.194], and EEG [F (2.977, 92.291) = 5.415; *p* = 0.002; *η* ^2^ = 0.149]. These results indicate that PSD varies across different fNIRS‐i, fNIRS‐e, and EEG channels. A significant interaction between condition and channels were also found in the case of EEG [F (3.124, 96.843) = 3.614; *p* = 0.015; *η* ^2^ = 0.104]. However, this interaction and the effect of channels in EEG and fNIRS‐e will not be further examined in this study, as our focus is on fNIRS‐i for localization topography due to its superior spatial resolution. A cortical topographical visualization of the PSD of fNIRS‐i results around 0.33 Hz is depicted in Figure 3a, which illustrates paired t‐tests (FDR corrected for 126 comparisons: 3 conditions × 42 channels) between conditions across all fNIRS‐i channels.

### 3.3 Phase coherence

The coherence of the different data signals with the auditory stimulation signal (0.33 Hz) is shown in Figure 2b. The repeated‐measures ANOVA showed a main effect of condition on phase coherence with the auditory stimulation signal (0.33 Hz) for all the recorded signals (Table 2).

**TABLE 2:** Statistical results from the coherence analysis of the different data signals with the auditory stimulation signal (0.33 Hz).

Signal	*Df*	*F*	*p*	*η* ^*2*^
SCR	1.850, 53.663	20.345	<0.001	0.412
HR	1.073, 36.953	34.316	<0.001	0.542
PPG	1.172, 33.978	22.453	<0.001	0.436
RSP	1.207, 34.998	25.375	<0.001	0.467
PTT	1.155, 33.486	14.950	<0.001	0.340
fNIRS‐e	1.330, 38.581	25.282	<0.001	0.466
fNIRS‐i	1.307, 37.900	31.664	<0.001	0.522
EEG	1.424, 41.292	88.927	<0.001	0.754

*Note*: Post hoc contrast analysis (FDR corrected for 24 comparisons: 3 conditions × 8 signals) revealed that coherence between SCR‐0.33 Hz was higher for the active and passive conditions as compared with the baseline [t (1, 29) = 6.382, 5.171, respectively; *p*_(FDR)_ < 0.001 in both cases]. Coherence between HR‐0.33 Hz, PPG‐0.33 Hz, RSP‐0.33 Hz, PTT‐0.33 Hz, fNIRS_e_‐0.33 Hz, and fNIRS_i_‐0.33 Hz were greater for the active as compared with the passive condition [t (1, 29) = 5.697, 4.868, 4.881, 3.924, 5.404, and 6.323, respectively; *p*_(FDR)_ < 0.001 in all cases], as well as for the active as compared with the baseline condition [t (1, 29) = 6.417, 4.872, 5.399, 3.991, 5.210, and 5.644, respectively; *p*_(FDR)_ < 0.001 in all cases]. Coherence between EEG‐0.33 Hz was higher for the active and passive conditions as compared with the baseline [t (1, 29) = 10.236, 4.298, respectively; *p*_(FDR)_ < 0.001 in both cases], as well as for the active as compared with the passive condition [t (1, 29) = 9.768; *p*_(FDR)_ < 0.001]. Altogether, these results indicate a greater consistency of the phase difference between all recorded signals and the auditory stimulation during the active condition. Abbreviations are specified in Table 1.

Additional repeated‐measures ANOVA with channels as an added factor found a main effect of channels on phase coherence with the auditory stimulation signal (0.33 Hz) for fNIRS‐i [F (13.698, 397.236) = 1.895; *p* < 0.001; *η* ^2^ = 0.061] and EEG [F (2.800, 81.214) = 6.923; *p* < 0.001; *η* ^2^ = 0.193]. Significant interactions between conditions and channels were also found in fNIRS‐i [F (16.654, 482.971) = 2.070; *p* = 0.008; *η* ^2^ = 0.067] and EEG [F (4.125, 120.204) = 4.429; *p* = 0.002; *η* ^2^ = 0.132]. These results indicate that coherence with the auditory stimulation signal (0.33 Hz) varies across fNIRS‐i and EEG channels, but only under specific conditions. Figure 3b displays paired t‐tests (FDR corrected for 126 comparisons: 3 conditions × 42 channels) comparing conditions for the coherence between the HbO signal of all fNIRS‐i channels and the auditory stimulation signal at 0.33 Hz, providing a topographical visualization of the coherence results on the cortical surface.

### 3.4 Phase angle

**FIGURE 4:**
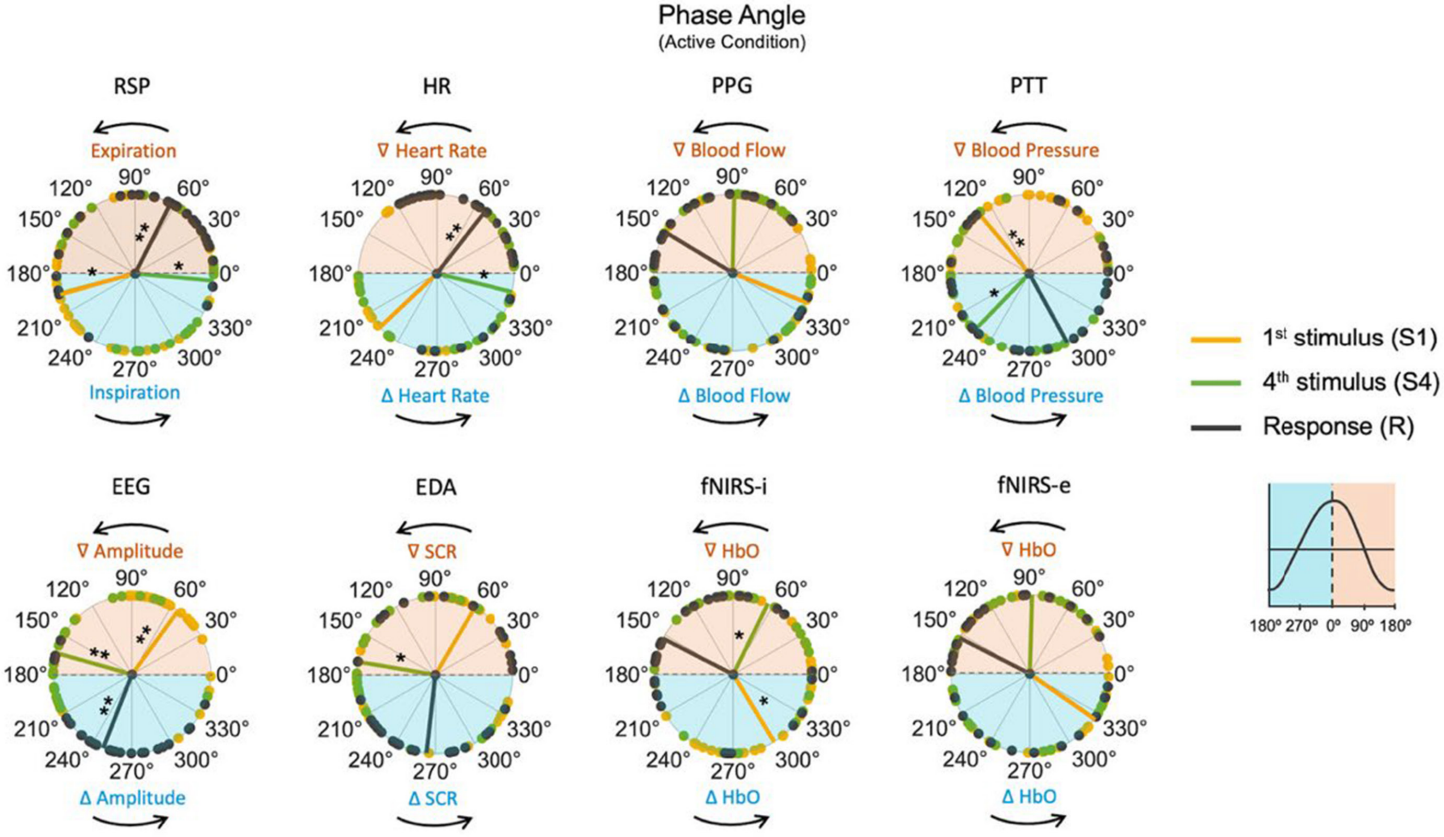
Circular representations of phase angles distribution in the active condition for each processed signal and type of driver: S1 (yellow), S4 (green), and R (black). Colored lines inside the circles represent the mean phase angle between participants for each type of driver. Each point around the circles represents a participant (*n* = 30). The legend box on the right shows the linear development of the signal equivalent to the circular phase angles, dividing the increasing phase (light blue color) from the decreasing phase (light peach color). Arrows above and below the circles indicate the direction of the signal's flow (counterclockwise). Angles are shown in degrees. Significant unimodal clustering between participants around the mean for each type of driver is specified with an asterisk for *p*_(FDR)_ < 0.05 and two asterisks for *p*_(FDR)_ < 0.01. EDA, electrodermal activity; EEG, electroencephalographic activity; fNIRS‐e, extracerebral HbO; fNIRS‐i, intracerebral/cortical HbO; HbO, oxyhemoglobin; HR, heart rate; PPG, peripheral pulse; PTT, pulse transit time; RSP, respiratory effort; SCR, skin conductance response.

The circular representations of the phase angles in the active condition for each processed signal and type of driver (S1, S4, and R) are shown in Figure 4. Rayleigh's test revealed significant unimodal clustering among participants (non‐uniform circular distribution) around a specific mean angle direction (θ) for the signals specified in Table 3 (FDR correction applied to 24 comparisons: 3 types of drivers × 8 signals). These results indicate that for certain signals, specific phase angles were consistently prioritized across participants at the onsets of stimuli and responses.

**TABLE 3:** Statistically significant results from the Rayleigh's test (*n* = 30).

Signal	Driver	θ	*SD*	*z*	*p*_(*FDR*)_
RSP	S1	195°	80°	0.378	0.034
	S4	355°	79°	0.383	0.033
	R	63°	63°	0.544	<0.001
HR	S4	347°	82°	0.363	0.037
	R	52°	69°	0.481	0.003
PTT	S1	130°	71°	0.461	0.005
	S4	226°	82°	0.362	0.037
EEG	S1	54°	46°	0.729	<0.001
	S4	164°	47°	0.718	<0.001
	R	249°	45°	0.739	<0.001
SCR	S4	170°	81°	0.364	0.037
fNIRS‐i	S1	301°	78°	0.399	0.026
	S4	64°	83°	0.350	0.044

*Note*: The remaining abbreviations are specified in Table 1.

Abbreviations: R, response; S1, first stimulus of the trial; S4, fourth stimulus of the trial; SD, standard deviation, in degrees; *z*, rayleigh's test coefficient; θ, mean angle direction, expressed in degrees.

A mixed‐effects model was applied to all recorded signals. FDR correction was applied to 48 comparisons, resulting from 3 types of drivers × 8 signals × 2 transformed phase angles (sine and cosine). Type of trial showed a clear association with reaction time, with longer reaction times for incongruent trials (*t* = 17.489, *p*_(FDR)_ < 0.001). Significant associations were also identified between reaction time and the sine‐transformed phase angle of the response (R) in RSP (*t* = 2.864, *p*_(FDR)_ = 0.038; shorter RT when closer to 270°), the cosine‐transformed phase angle of S1 in HR (*t* = −2.654, *p*_(FDR)_ = 0.048; shorter RT when closer to 0°), the sine‐transformed phase angle of S4 in HR (*t* = −3.744, *p*_(FDR)_ = 0.003; shorter RT when closer to 90°), the cosine‐transformed phase angle of S1 in EEG (*t* = 3.042, *p*_(FDR)_ = 0.028; shorter RT when closer to 180°), the sine and cosine transformed phase angle of S4 in EEG (*t* = 2.620, 4.716; *p*_(FDR)_ = 0.040, <0.001, respectively; shorter RT when closer to 180° and 270°, respectively), the cosine‐transformed phase angle of the response in EEG (*t* = 2.643, *p*_(FDR)_ = 0.040; shorter RT when closer to 180°) and the sine‐transformed phase angle of S1 in fNIRS‐i (*t* = −2.663, *p*_(FDR)_ = 0.040; shorter RT when closer to 90°). Random effects for participant indicated significant variability in the intercept of reaction time (participant: Var = 11,619, SD = 108, in milliseconds).

Overall, these results indicate that longer reaction times were observed when the RSP phase angle was near 90° at response onset, approximating the mean phase angle of 63° across participants. Similarly, longer reaction times occurred when the fNIRS‐i phase angle was near 270° at S1 onset, close to the group mean of 301°. In contrast, shorter reaction times were associated with an EEG phase angle around 180° at S4 onset, which approximates the group mean phase angle of 164°. Additionally, shorter reaction times were noted when the HR phase angle was near 90° at S4 onset, although this did not align with the mean phase angle of 347° across participants.

**FIGURE 5:**
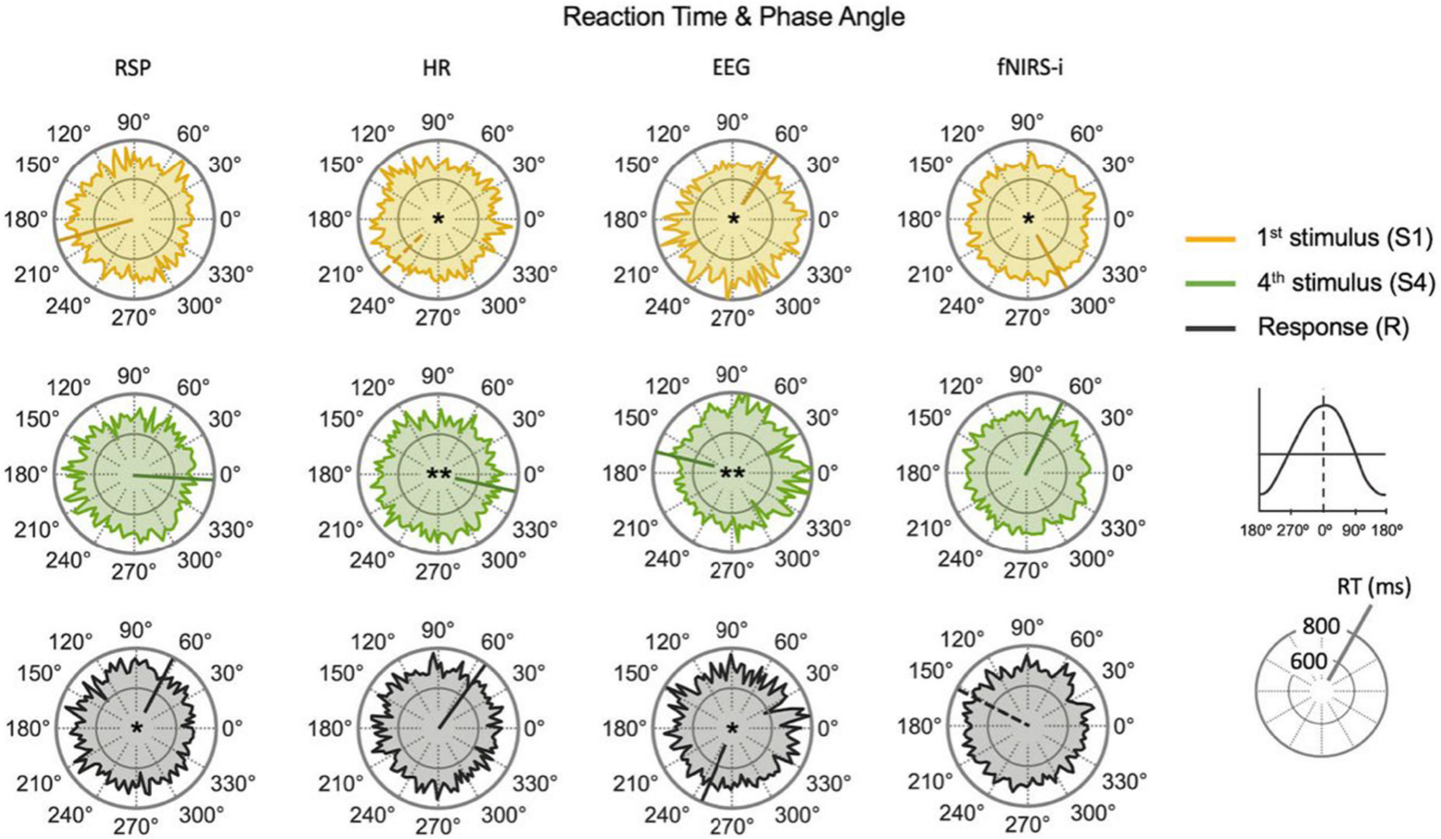
Circular representation of the relationship between reaction time (RT) and phase angle across all trials and participants for the three types of drivers: S1 (yellow), S4 (green), and R (black). Radial axis indicates RT in milliseconds (ms), ranging from 600 to 800 (right‐bottom legend). RT were averaged every three degrees to facilitate smoother visualization. Inside the circles, a solid or dashed radial line represents the mean phase angle across participants, indicating a significant or non‐significant cluster effect, respectively. The figure in the right‐middle legend displays the linear development of the signal equivalent to the circular phase angles. Significant associations between RT and sine/cosine transformed phase angle, identified through mixed‐effects model analysis, are indicated at the center of the circles with an asterisk for *p*_(FDR)_ < 0.05 and two asterisks for *p*_(FDR)_ < 0.01. EEG, electroencephalographic activity; fNIRS‐i, intracerebral/cortical HbO; HR, heart rate; RSP, respiratory effort.

Figure 5 displays the average RT computed for all trials and participants at every phase angle (360°). Only signals that show significant associations between reaction time and sine/cosine‐transformed phase angles for S1, S4, or R are displayed.

## 4 DISCUSSION

This study aimed to shed light on the complex dynamics of brain and body rhythms in response to rhythmic auditory stimulation and their behavioral implications, distinguishing between passive and active sensory encoding processes. Our findings indicate that active engagement with rhythmic auditory stimuli holistically entrains various bodily oscillations to the external rhythm, offering insights into the intricate interplay between bodily and environmental rhythms and their implications for sensorimotor performance.

### 4.1 Power spectral density

The PSD results revealed a pronounced peak at 0.33 Hz across all central and autonomic nervous system signals recorded during the active condition, corresponding to the frequency of auditory stimulation. Statistical analysis confirmed significant differences in PSD between the active condition and both passive and baseline conditions, except for PPG, which did not exhibit significant differences between the active and passive conditions. These results align with existing literature on neural entrainment as a mechanism of selective attention (Lakatos et al., 2008, 2019; Obleser & Kayser, 2019), extending this phenomenon to cortical oxygenation levels and autonomic responses during attentional engagement in sensorimotor cognitive tasks.

Additionally, the PSD of the EEG at 0.33 Hz was higher in the passive condition compared to the baseline condition, indicating neural entrainment to sensory stimulation even in the absence of conscious attentional effort, consistent with previous reports (Criscuolo, Schwartze, Henry, et al., 2023; Criscuolo, Schwartze, Prado, et al., 2023). However, no entrainment was observed for cortical oxygenation levels during the passive condition compared to the baseline condition, suggesting that passive entrainment may require lower metabolic brain resources.

Concerning the cortical topography of the PSD results at the frequency of auditory stimulation, measured through fNIRS‐i, the findings demonstrated a greater amplitude of cortical HbO at 0.33 Hz in the active condition compared to both passive and baseline conditions, particularly in the inferior frontal gyrus (IFG), superior temporal gyrus (STG), and inferior parietal lobule (IPL), which are cortical regions associated with keeping the meter in musical contexts (Vuust et al., 2006).

These PSD results suggest a generalized entrainment across various bodily oscillations as a means of actively engaging with rhythmic auditory stimulation, while the entrainment of cortical oxygenation levels exhibits a localized effect in brain regions specialized for auditory processing.

### 4.2 Phase coherence

Consistent with the PSD results, the coherence analysis provided additional evidence of increased entrainment to auditory stimulation for all recorded signals during the active condition, in comparison to both passive and baseline conditions. In this case, alongside EEG, SCR also demonstrated heightened synchronization with incoming auditory inputs during the passive condition compared to the baseline. Interestingly, no significant differences were observed in SCR coherence with auditory inputs between the active and passive conditions. Given that phasic EEG‐EDA responses have been shown to represent salience‐dependent reactions to incoming stimuli as part of the orienting or defensive reflex, irrespective of attentional engagement (Muñoz‐Caracuel et al., 2021; Sokolov, 1990), these findings could be interpreted as indicative of a subtle form of underlying communication between the CNS and ANS occurring in the context of rhythmic stimulation with variable sequence presentation.

The cortical topography of coherence results with auditory stimulation, in contrast to PSD cortical topography, revealed greater coherence of HbO levels with the auditory stimulation across the entire recorded cortical surface during the active condition, rather than at more specialized cortical regions. This contrast could reflect that while the oscillatory dynamics of cortical HbO signal amplitude at specific frequencies are more dependent on neurovascular coupling (Muñoz et al., 2023), the underlying oscillatory dynamics of HbO cortical levels (regardless of their amplitude levels) might be more influenced by peripheral cardiovascular inputs. This interpretation is supported by the concomitant higher coherence with auditory stimulation found in the active condition for extracerebral HbO flow (fNIRS‐e), measured through the fNIRS short channels, and the interaction effect between condition and channels observed in the PSD of fNIRS‐e.

Considering the results from both PSD and coherence analyses, our findings indicate an entrainment effect on central nervous system (CNS) and autonomic nervous system (ANS) physiological rhythms during active engagement with rhythmic auditory stimuli, compared to passive listening and baseline conditions. This expands existing evidence on neural entrainment (Henry et al., 2014; Lakatos et al., 2008; Obleser & Kayser, 2019; Schroeder & Lakatos, 2009) and respiratory entrainment (Haas et al., 1986; Wilke et al., 1975) to external sensory inputs, demonstrating a systemic effect that encompasses the entire body. However, considering the debate on the definition and mechanisms of entrainment in the field of neuroscience (Haegens, 2020; Meyer et al., 2020), our interpretation of the results should be cautious, as we cannot exclude the possibility that different or additional mechanisms (e.g., a series of evoked responses) might contribute to the observed rhythmic tracking. Further research is required to address this aspect in this evolving field.

### 4.3 Phase angle

After elucidating systemic neurophysiological entrainment to auditory stimulation during the active condition, two other questions remain: (i) Do specific oscillatory phases consistently align across participants with the onset of auditory stimulation or task responses? (ii) If so, what are the behavioral implications or functional advantages of phase‐specific entrainment across participants?

Regarding the first question (i), the EEG phase angle analysis revealed consistent occurrences of S1, S4, and R arrivals around the same oscillatory phases across participants. Notably, S4 closely aligns with the negative peak of the EEG oscillatory activity (180°). These findings are consistent with our previously reported Event‐Related Potentials (ERPs) results from this paradigm (Muñoz‐Caracuel et al., 2024), indicating that the EEG slow negative deflection commence at the cue stimulus arrival (S1) and reaches its negative peak just after the arrival of the target stimulus (S4). This oscillatory dynamic is interpreted as a combination of two related slow ERPs: Contingent Negative Variation (CNV), representing expectancy and anticipatory activity, and Post Imperative Negative Variation (PINV), representing information updating and baseline recovery (Muñoz‐Caracuel et al., 2024; Ruiz‐Martínez et al., 2022).

Other bodily oscillations also exhibited predominant oscillatory phases at the arrival of S1, S4, or R, albeit at different phase angles. RSP, for instance, consistent with previously reported findings (Johannknecht & Kayser, 2022; Perl et al., 2019), displayed a tendency to entrain the inspiration phase with the arrival of the cue stimuli (S1) and the expiration phase with the target stimuli (S4), finally exerting the motor response (R) during the expiration phase. HR showed a phase pattern similar to that of RSP, as expected from cardiorespiratory coupling through respiratory sinus arrhythmia (RSA) (Parviainen et al., 2022). While EDA (SCR) exhibited less uniform distribution across participants for S4 and R, it displayed a phase pattern similar to EEG, further supporting the interrelationship between these two signals (Lim et al., 1999; Martínez Vásquez et al., 2023). fNIRS‐i and PTT also demonstrated uniformity across participants at the arrival of auditory stimuli, albeit at different phase angles. Finally, PPG and fNIRS‐e did not exhibit significant uniformity of phase angles, although their phase angle distribution illustrated a very similar pattern, consistent with the expected similarity for blood flow measures.

In our final exploration, to answer the second question (ii) we investigated the potential behavioral implications (adaptive function) of phase‐specific entrainment across participants by examining the association between reaction times (RT) and prevalent phases angles. Association between accuracy and prevalent phases angles could not be analyzed due to the marginal number of errors in the sample. Neural entrainment across participants to the target stimuli (S4) onset, measured from EEG slow waves, was associated with reduced reaction times, providing further support for the functional advantages of neural entrainment to external inputs in adaptive sensorimotor behavior (Nozaradan et al., 2016; Stefanics et al., 2010). However, the association of other bodily oscillation phases with reaction times was not found for S4 arrival or pointed in different directions, as in the case of HR, where shorter reaction times were found for non‐prevalent phase angles across participants at S4 arrival. Interestingly, respiration showed a significant association between prevalent phase angles at the response onset (R) and reaction time, with longer reaction times for the most prevalent respiratory phase (expiration) at R onset. Although the influence of respiratory cycle on reaction time have been previously demonstrated (Johannknecht & Kayser, 2022), these contradictory results between EEG and respiration with reaction time could be interpreted in the context of mixed speed‐accuracy task demands, where neural entrainment to target stimuli could provide speed performance advantages, while respiration prevalent states at the response onset could act as an inhibitory servomechanism to prevent unnecessary errors in favor of more accurate responses.

These findings indicate that sensorimotor engagement with rhythmic auditory stimuli holistically entrains bodily oscillations to the rhythm in the frequency domain, though at different points in the phase domain. Interestingly, individuals demonstrate an inherent predisposition to entrain to specific phase angles of various physiological oscillations at the onsets of stimuli or responses. This suggests a complex, multifaceted inferential process aimed at enhancing sensory coding and facilitating adaptive speed‐accuracy behavior.

### 4.4 Predictive processing

From a predictive processing perspective, the generalized entrainment found of both ANS and CNS rhythms to external auditory stimuli during the active condition, along with the phase angles prioritized across participants, may reflect a predictive timing function. This function could involve higher hierarchical levels generating internal representations of the external rhythm (Meyer et al., 2020) and preparing various physiological subsystems for optimal sensorimotor performance. In other words, active engagement in sensorimotor tasks might involve top‐down temporal predictions from higher cortical levels (neural entrainment) that propagate through different hierarchical levels to various physiological subsystems (Arnal & Giraud, 2012; Engel et al., 2001; Parr et al., 2022; Pezzulo et al., 2015; Vuust & Witek, 2014). These predictions could structure activity patterns, potentially optimizing information processing and enhancing behavioral performance (Kluger et al., 2021; Nozaradan et al., 2016). By orchestrating these temporal predictions, the brain might ensure that sensory inputs are processed at optimal moments, thereby reducing cognitive load and enabling more efficient and accurate motor responses.

Additionally, the observed increase in RT during preferred respiratory phases at the response onset could be interpreted within the predictive processing framework as indicating that participants are actively seeking expected (preferred) body states that serve an adaptive function in goal‐directed behavior (Allen et al., 2023; Brændholt et al., 2023; Criscuolo et al., 2022; Kluger et al., 2021; Parr et al., 2022). In this case, participants' expectations (predictions) of congruent S4 (80% of the time) might occasionally elicit rapid automatic responses, leading to unnecessary errors in response to incongruent stimuli. Thus, adaptive (error‐free) behavioral performance might benefit from predictive processes that actively entrain specific bodily oscillation phases acting as inhibitory servomechanisms at the moment of response. This could facilitate longer reaction times, thereby enhancing accuracy and cognitive control in sensorimotor tasks.

Finally, the interplay between predictive coding and bodily entrainment illustrates a dynamic interaction where the central nervous system coordinates with the autonomic nervous system to optimize sensorimotor responses to environmental demands.

## 5 CONCLUSIONS

The present study provides empirical neurophysiological evidence of systemic bodily entrainment to behaviorally relevant rhythmic auditory stimulation. This highlights the interconnectedness of the central and autonomic nervous systems in facilitating adaptive sensorimotor responses to environmental demands and suggests a potential manifestation of embodied predictive processing.

## AUTHOR CONTRIBUTIONS

M.M.‐C. and C.M.G. conceived and designed the experiment. M.M.‐C. and V.M. conducted the experiment. F.J.R.‐M. contributed to the EEG data preprocessing. M.M.‐C. performed the data analysis and wrote the manuscript. A.J.V.M. contributed to conceptualization. C.M.G. supervised data analysis and reviewed the manuscript. All authors revised the manuscript and approved the final version and agree to be accountable for all aspects of the work in ensuring that questions related to the accuracy or integrity of any part of the work are appropriately investigated and resolved. All persons designated as authors qualify for authorship, and all those who qualify for authorship are listed.

## FUNDING INFORMATION

This work was supported by the Spanish Agencia Estatal de Investigación (AEI) (PID2022‐139151OB‐I00) (FEDER funds), and the Consejería de Innovación, Ciencia y Empresa of the Junta de Andalucía (P20_00537).

## CONFLICT OF INTEREST STATEMENT

The authors declare no competing interests.

## ETHICS STATEMENT

The study was approved by the Bioethical Committee of the Junta de Andalucía. All participants provided written informed consent prior to participation, following the Helsinki Protocol.

## Data Availability

The data that support the findings of this study are available from the corresponding author (mmunoz41@us.es) upon reasonable request.

## References

[phy270079-bib-0001] Allen, M. , & Friston, K. J. (2018). From cognitivism to autopoiesis: Towards a computational framework for the embodied mind. Synthese, 195(6), 2459–2482. 10.1007/S11229-016-1288-5/FIGURES/4 29887647 PMC5972168

[phy270079-bib-0002] Allen, M. , Varga, S. , & Heck, D. H. (2023). Respiratory rhythms of the predictive mind. Psychological Review, 130(4), 1066–1080. 10.1037/REV0000391 35980689

[phy270079-bib-0003] Arnal, L. H. , & Giraud, A. L. (2012). Cortical oscillations and sensory predictions. Trends in Cognitive Sciences, 16(7), 390–398. 10.1016/J.TICS.2012.05.003 22682813

[phy270079-bib-0004] Benedek, M. , & Kaernbach, C. (2010). A continuous measure of phasic electrodermal activity. Journal of Neuroscience Methods, 190(1), 80–91. 10.1016/J.JNEUMETH.2010.04.028 20451556 PMC2892750

[phy270079-bib-0005] Brændholt, M. , Kluger, D. S. , Varga, S. , Heck, D. H. , Gross, J. , & Allen, M. G. (2023). Breathing in waves: Understanding respiratory‐brain coupling as a gradient of predictive oscillations. Neuroscience & Biobehavioral Reviews, 152, 105262. 10.1016/J.NEUBIOREV.2023.105262 37271298

[phy270079-bib-0006] Brigadoi, S. , Ceccherini, L. , Cutini, S. , Scarpa, F. , Scatturin, P. , Selb, J. , Gagnon, L. , Boas, D. A. , & Cooper, R. J. (2014). Motion artifacts in functional near‐infrared spectroscopy: A comparison of motion correction techniques applied to real cognitive data. NeuroImage, 85, 181–191. 10.1016/J.NEUROIMAGE.2013.04.082 23639260 PMC3762942

[phy270079-bib-0007] Charalambous, E. , & Djebbara, Z. (2023). On natural attunement: Shared rhythms between the brain and the environment. Neuroscience & Biobehavioral Reviews, 155, 105438. 10.1016/J.NEUBIOREV.2023.105438 37898445

[phy270079-bib-0008] Clark, A. (2013). Whatever next? Predictive brains, situated agents, and the future of cognitive science. Behavioral and Brain Sciences, 36(3), 181–204. 10.1017/S0140525X12000477 23663408

[phy270079-bib-0009] Cooper, R. J. , Selb, J. , Gagnon, L. , Phillip, D. , Schytz, H. W. , Iversen, H. K. , Ashina, M. , & Boas, D. A. (2012). A systematic comparison of motion artifact correction techniques for functional near‐infrared spectroscopy. Frontiers in Neuroscience, 6, 32692. 10.3389/FNINS.2012.00147/BIBTEX PMC3468891 23087603

[phy270079-bib-0010] Criscuolo, A. , Schwartze, M. , Henry, M. J. , Obermeier, C. , & Kotz, S. A. (2023). Individual neurophysiological signatures of spontaneous rhythm processing. NeuroImage, 273, 120090. 10.1016/j.neuroimage.2023.120090 37028735

[phy270079-bib-0011] Criscuolo, A. , Schwartze, M. , & Kotz, S. A. (2022). Cognition through the lens of a body–brain dynamic system. Trends in Neurosciences, 45(9), 667–677. 10.1016/J.TINS.2022.06.004 35810022

[phy270079-bib-0012] Criscuolo, A. , Schwartze, M. , Prado, L. , Ayala, Y. , Merchant, H. , & Kotz, S. A. (2023). Macaque monkeys and humans sample temporal regularities in the acoustic environment. Progress in Neurobiology, 229, 102502. 10.1016/J.PNEUROBIO.2023.102502 37442410

[phy270079-bib-0013] Engel, A. K. , Fries, P. , & Singer, W. (2001). Dynamic predictions: Oscillations and synchrony in top–down processing. Nature Reviews Neuroscience, 2(10), 704–716. 10.1038/35094565 11584308

[phy270079-bib-0014] Friston, K. (2010). The free‐energy principle: A unified brain theory? Nature Reviews Neuroscience, 11(2), 127–138. 10.1038/nrn2787 20068583

[phy270079-bib-0015] Glass, L. (2001). Rhythmic processes in physiology. Nature, 410, 277–284. 11258383 10.1038/35065745

[phy270079-bib-0016] Greenfield, M. D. , Honing, H. , Kotz, S. A. , & Ravignani, A. (2021). Synchrony and rhythm interaction: From the brain to behavioural ecology. Philosophical Transactions of the Royal Society B, 376(1835), 20200324. 10.1098/RSTB.2020.0324 PMC8384058 34420379

[phy270079-bib-0017] Haas, F. , Distenfeld, S. , & Axen, K. (1986). Effects of perceived musical rhythm on respiratory pattern. Journal of Applied Physiology, 61(3), 1185–1191. 10.1152/JAPPL.1986.61.3.1185 3759758

[phy270079-bib-0018] Haegens, S. (2020). Entrainment revisited: A commentary on. Language, Cognition and Neuroscience, 35(9), 1119–1123. 10.1080/23273798.2020.1758335 33718510 PMC7954236

[phy270079-bib-0019] Henry, M. J. , Herrmann, B. , & Obleser, J. (2014). Entrained neural oscillations in multiple frequency bands comodulate behavior. Proceedings of the National Academy of Sciences of the United States of America, 111(41), 14935–14940. 10.1073/pnas.1408741111 25267634 PMC4205645

[phy270079-bib-0020] Herrero, J. L. , Khuvis, S. , Yeagle, E. , Cerf, M. , & Mehta, A. D. (2018). Breathing above the brain stem: Volitional control and attentional modulation in humans. Journal of Neurophysiology, 119(1), 145–159. 10.1152/jn.00551.2017 28954895 PMC5866472

[phy270079-bib-0021] Hohwy, J. (2013). The Predictive Mind. The Predictive Mind. 10.1093/ACPROF:OSO/9780199682737.001.0001

[phy270079-bib-0022] Huppert, T. J. , Diamond, S. G. , Franceschini, M. A. , & Boas, D. A. (2009). HomER: A review of time‐series analysis methods for near‐infrared spectroscopy of the brain. Applied Optics, 48(10), D280–D298. 10.1364/AO.48.00D280 19340120 PMC2761652

[phy270079-bib-0023] Jammalamadaka, S. R. , & SenGupta, A. (2001). Series on multivariate analysis: Topics in circular statistics. In World Scientific (Vol. 5). World scientific pub Co Inc.

[phy270079-bib-0024] Johannknecht, M. , & Kayser, C. (2022). The influence of the respiratory cycle on reaction times in sensory‐cognitive paradigms. Scientific Reports, 12(1), 1–17. 10.1038/s41598-022-06364-8 35173204 PMC8850565

[phy270079-bib-0025] Kluger, D. S. , Balestrieri, E. , Busch, N. A. , & Gross, J. (2021). Respiration aligns perception with neural excitability. eLife, 10, 1–19. 10.7554/eLife.70907 PMC8763394 34904567

[phy270079-bib-0026] Kotz, S. A. , Ravignani, A. , & Fitch, W. T. (2018). The evolution of rhythm processing. Trends in Cognitive Sciences, 22(10), 896–910. 10.1016/J.TICS.2018.08.002 30266149

[phy270079-bib-0027] Lakatos, P. , Gross, J. , & Thut, G. (2019). A new unifying account of the roles of neuronal entrainment. Current Biology, 29(18), R890–R905. 10.1016/J.CUB.2019.07.075 31550478 PMC6769420

[phy270079-bib-0028] Lakatos, P. , Karmos, G. , Mehta, A. D. , Ulbert, I. , & Schroeder, C. E. (2008). Entrainment of neuronal oscillations as a mechanism of attentional selection. Science, 320(5872), 110–113. 10.1126/SCIENCE.1154735/SUPPL_FILE/LAKATOS.SOM.PDF 18388295

[phy270079-bib-0029] Lakatos, P. , Musacchia, G. , O'Connel, M. N. , Falchier, A. Y. , Javitt, D. C. , & Schroeder, C. E. (2013). The spectrotemporal filter mechanism of auditory selective attention. Neuron, 77(4), 750–761. 10.1016/j.neuron.2012.11.034 23439126 PMC3583016

[phy270079-bib-0030] Lim, C. L. , Gordon, E. , Rennie, C. , Wright, J. J. , Bahramali, H. , Li, W. M. , Clouston, P. , & Morris, J. G. L. (1999). Dynamics of SCR, EEG, and ERP activity in an oddball paradigm with short interstimulus intervals. Psychophysiology, 36(5), 543–551. 10.1017/S0048577299971226 10442022

[phy270079-bib-0031] Maric, V. , Ramanathan, D. , & Mishra, J. (2020). Respiratory regulation & interactions with neuro‐cognitive circuitry. Neuroscience and Biobehavioral Reviews, 112, 95–106. 10.1016/j.neubiorev.2020.02.001 32027875 PMC10092293

[phy270079-bib-0032] Martínez Vásquez, D. A. , Posada‐Quintero, H. F. , & Rivera Pinzón, D. M. (2023). Mutual information between EDA and EEG in multiple cognitive tasks and sleep deprivation conditions. Behavioral Science, 13(9), 707. 10.3390/BS13090707 PMC10525564 37753985

[phy270079-bib-0033] Meyer, L. , Sun, Y. , & Martin, A. E. (2020). Synchronous, but not entrained: Exogenous and endogenous cortical rhythms of speech and language processing. Language, Cognition and Neuroscience, 35(9), 1089–1099. 10.1080/23273798.2019.1693050

[phy270079-bib-0062] Molavi, B. , & Dumont, G. A. (2012). Wavelet‐based motion artifact removal for functional near‐infrared spectroscopy. Physiological Measurement, 33(2), 259–270. 10.1088/0967-3334/33/2/259 22273765

[phy270079-bib-0035] Muñoz, V. , Muñoz‐Caracuel, M. , Angulo‐Ruiz, B. Y. , & Gómez, C. M. (2023). Neurovascular coupling during auditory stimulation: Event‐related potentials and fNIRS hemodynamic. Brain Structure and Function, 228(8), 1943–1961. 10.1007/s00429-023-02698-9 37658858 PMC10517045

[phy270079-bib-0036] Muñoz‐Caracuel, M. , Muñoz, V. , Ruíz‐Martínez, F. J. , Di Domenico, D. , Brigadoi, S. , & Gómez, C. M. (2021). Multivariate analysis of the systemic response to auditory stimulation: An integrative approach. Experimental Physiology, 106(4), 1072–1098. 10.1113/EP089125 33624899

[phy270079-bib-0037] Muñoz‐Caracuel, M. , Muñoz, V. , Ruiz‐Martínez, F. J. , Vázquez Morejón, A. J. , & Gómez, C. M. (2024). Systemic neurophysiological signals of auditory predictive coding. Psychophysiology, 61(6), 1–24. 10.1111/psyp.14544 38351668

[phy270079-bib-0038] Mütze, H. , Kopiez, R. , & Wolf, A. (2020). The effect of a rhythmic pulse on the heart rate: Little evidence for rhythmical ‘entrainment’ and ‘synchronization’. Musicae Scientiae, 24(3), 377–400. 10.1177/1029864918817805

[phy270079-bib-0039] Nozaradan, S. , Peretz, I. , & Keller, P. E. (2016). Individual differences in rhythmic cortical entrainment correlate with predictive behavior in sensorimotor synchronization. Scientific Reports, 6, 1–12. 10.1038/srep20612 26847160 PMC4742877

[phy270079-bib-0040] Obleser, J. , & Kayser, C. (2019). Neural entrainment and attentional selection in the listening brain. Trends in Cognitive Sciences, 23(11), 913–926. 10.1016/j.tics.2019.08.004 31606386

[phy270079-bib-0041] Owens, A. P. , Allen, M. , Ondobaka, S. , & Friston, K. J. (2018). Interoceptive inference: From computational neuroscience to clinic. Neuroscience and Biobehavioral Reviews, 90, 174–183. 10.1016/j.neubiorev.2018.04.017 29694845

[phy270079-bib-0042] Parr, T. , Pezzulo, G. , & Friston, K. J. (2022). Active inference: The free energy principle in mind, brain, and behavior. The MIT Press. 10.7551/MITPRESS/12441.001.0001

[phy270079-bib-0043] Parviainen, T. , Lyyra, P. , & Nokia, M. S. (2022). Cardiorespiratory rhythms, brain oscillatory activity and cognition: Review of evidence and proposal for significance. Neuroscience and Biobehavioral Reviews, 142, 104908. 10.1016/j.neubiorev.2022.104908 36220367

[phy270079-bib-0044] Peirce, J. W. (2009). Generating stimuli for neuroscience using PsychoPy. Frontiers in Neuroinformatics, 2, 1–8. 10.3389/neuro.11.010.2008 PMC2636899 19198666

[phy270079-bib-0045] Penzel, T. , Porta, A. , Stefanovska, A. , & Wessel, N. (2017). Recent advances in physiological oscillations. Physiological Measurement, 38(5), E1–E7. 10.1088/1361-6579/AA6780 28452338

[phy270079-bib-0046] Perl, O. , Ravia, A. , Rubinson, M. , Eisen, A. , Soroka, T. , Mor, N. , Secundo, L. , & Sobel, N. (2019). Human non‐olfactory cognition phase‐locked with inhalation. Nature Human Behaviour, 3(5), 501–512. 10.1038/s41562-019-0556-z 31089297

[phy270079-bib-0047] Pezzulo, G. , Rigoli, F. , & Friston, K. (2015). Active inference, homeostatic regulation and adaptive behavioural control. Progress in Neurobiology, 134, 17–35. 10.1016/j.pneurobio.2015.09.001 26365173 PMC4779150

[phy270079-bib-0048] Pikovsky, A. , Rosenblum, M. , & Kurths, J. (2001). Synchronization: A universal concept in nonlinear sciences. Cambridge University Press. 10.1017/CBO9780511755743

[phy270079-bib-0049] Rapp, P. E. (1987). Why are so many biological systems periodic? Progress in Neurobiology, 29, 261–273. 10.1016/0301-0082(87)90023-2 3299493

[phy270079-bib-0050] Ruiz‐Martínez, F. J. , Morales‐Ortiz, M. , & Gómez, C. M. (2022). Late N1 and postimperative negative variation analysis depending on the previous trial history in paradigms of increasing auditory complexity. Journal of Neurophysiology, 127(5), 1240–1252. 10.1152/JN.00313.2021 35389770

[phy270079-bib-0051] Scholkmann, F. , Spichtig, S. , Muehlemann, T. , & Wolf, M. (2010). How to detect and reduce movement artifacts in near‐infrared imaging using moving standard deviation and spline interpolation. Physiological Measurement, 31(5), 649–662. 10.1088/0967-3334/31/5/004 20308772

[phy270079-bib-0052] Schroeder, C. E. , & Lakatos, P. (2009). Low‐frequency neuronal oscillations as instruments of sensory selection. Trends in Neurosciences, 32(1), 9–18. 10.1016/j.tins.2008.09.012 19012975 PMC2990947

[phy270079-bib-0053] Seth, A. K. , & Friston, K. J. (2016). Active interoceptive inference and the emotional brain. Philosophical Transactions of the Royal Society, B: Biological Sciences, 371(1708), 20160007. 10.1098/RSTB.2016.0007 PMC5062097 28080966

[phy270079-bib-0054] Sokolov, E. N. (1990). The orienting response, and future directions of its development. The Pavlovian Journal of Biological Science, 25(3), 142–150. 10.1007/BF02974268 2287527

[phy270079-bib-0055] Stefanics, G. , Hangya, B. , Hernádi, I. , Winkler, I. , Lakatos, P. , & Ulbert, I. (2010). Phase entrainment of human delta oscillations can mediate the effects of expectation on reaction speed. Journal of Neuroscience, 30(41), 13578–13585. 10.1523/JNEUROSCI.0703-10.2010 20943899 PMC4427664

[phy270079-bib-0056] Vuust, P. , Roepstorff, A. , Wallentin, M. , Mouridsen, K. , & Østergaard, L. (2006). It don't mean a thing… Keeping the rhythm during polyrhythmic tension, activates language areas (BA47). NeuroImage, 31(2), 832–841. 10.1016/J.NEUROIMAGE.2005.12.037 16516496

[phy270079-bib-0057] Vuust, P. , & Witek, M. A. G. (2014). Rhythmic complexity and predictive coding: A novel approach to modeling rhythm and meter perception in music. Frontiers in Psychology, 5, 1–14. 10.3389/fpsyg.2014.01111 25324813 PMC4181238

[phy270079-bib-0058] Wilke, J. T. , Lansing, R. W. , & Rogers, C. A. (1975). Entrainment of respiration to repetitive finger tapping. Physiological Psychology, 3(4), 345–349. 10.3758/BF03326838/METRICS

[phy270079-bib-0059] Xiong, L. , & Garfinkel, A. (2023). Are physiological oscillations physiological? Journal of Physiology, Advance online publication. 10.1113/JP285015 PMC13134434 37622389

[phy270079-bib-0060] Zelano, C. , Jiang, H. , Zhou, G. , Arora, N. , Schuele, S. , Rosenow, J. , & Gottfried, J. A. (2016). Nasal respiration entrains human limbic oscillations and modulates cognitive function. Journal of Neuroscience, 36(49), 12448–12467. 10.1523/JNEUROSCI.2586-16.2016 27927961 PMC5148230

[phy270079-bib-0061] Zion Golumbic, E. M. , Ding, N. , Bickel, S. , Lakatos, P. , Schevon, C. A. , McKhann, G. M. , Goodman, R. R. , Emerson, R. , Mehta, A. D. , Simon, J. Z. , Poeppel, D. , & Schroeder, C. E. (2013). Mechanisms underlying selective neuronal tracking of attended speech at a “cocktail party”. Neuron, 77(5), 980–991. 10.1016/J.NEURON.2012.12.037 23473326 PMC3891478

